# Overweight Women with Breast Cancer on Chemotherapy Have More Unfavorable Inflammatory and Oxidative Stress Profiles

**DOI:** 10.3390/nu12113303

**Published:** 2020-10-28

**Authors:** Letícia L. D. Santos, Isis D. D. Custódio, Alinne T. F. Silva, Izabella C. C. Ferreira, Eduarda C. Marinho, Douglas C. Caixeta, Adriele V. Souza, Renata R. Teixeira, Thaise G. Araújo, Nitin Shivappa, James R. Hébert, Carlos Eduardo Paiva, Foued S. Espíndola, Luiz Ricardo Goulart, Yara C. P. Maia

**Affiliations:** 1 Laboratory of Nanobiotechnology, Institute of Biotechnology, Federal University of Uberlândia, Uberlândia 38402-022, Brazil; leticialopesdantas@yahoo.com.br (L.L.D.S.); alinnetatianefaria@gmail.com (A.T.F.S.); izabellacostaferreira@gmail.com (I.C.C.F.); tgaraujo@ufu.br (T.G.A.); 2 Graduate Program in Health Sciences, Federal University of Uberlândia, Uberlândia 38400-902, Brazil; isisdanyelle@yahoo.com.br (I.D.D.C.); eduardacosta.marinho@gmail.com (E.C.M.); foued@ufu.br (F.S.E.); 3 Laboratory of Biochemistry and Molecular Biology, Institute of Biotechnology, Federal University of Uberlândia, Uberlândia 38405-302, Brazil; caixetadoug@gmail.com (D.C.C.); adriele_vds@hotmail.com (A.V.S.); rolandteixeira@yahoo.com (R.R.T.); 4 South Carolina Statewide Cancer Prevention and Control Program, University of South Carolina, Columbia, SC 29208, USA; shivappa@email.sc.edu (N.S.); jhebert@mailbox.sc.edu (J.R.H.); 5 Department of Epidemiology and Biostatistics, University of South Carolina, Columbia, SC 29208, USA; 6 Department of Nutrition, Connecting Health Innovations LLC, Columbia, SC 29201, USA; 7 Department of Clinical Oncology, Graduate Program in Oncology, Palliative Care and Quality of Life Research Group (GPQual), Pio XII Foundation—Barretos Cancer Hospital, Barretos 14784-400, Brazil; caredupai@gmail.com

**Keywords:** breast cancer, dietary total antioxidant capacity, dietary inflammatory index, chronic inflammation, oxidative stress, plasma biomarkers

## Abstract

Chronic inflammation and redox imbalance are strongly influenced by diet and nutritional status, and both are risk factors for tumor development. This prospective study aimed to explore the associations between inflammatory and antioxidant markers and nutritional status in women with breast cancer undergoing chemotherapy. The women were evaluated at three times: T0, after the infusion of the first cycle; T1, after infusion of the intermediate cycle; and T2, after the infusion of the last chemotherapy cycle. The consumption of antioxidant nutrients and the Total Dietary Antioxidant Capacity reduced between T0 and T2 and the Dietary Inflammatory Index scores increased throughout the chemotherapy. Blood samples taken at the end of the chemotherapy showed lower levels of glutathione reductase and reduced glutathione, with greater quantification of the transcripts for Interleukin-6 and Tumor Necrosis Factor α. It should be emphasized that the Total Dietary Antioxidant Capacity is lower and the Dietary Inflammatory Index is higher in the group of overweight patients at the end of the follow-up, besides showing lower levels of the redox status, especially the plasma levels of glutathione reductase (*p* = 0.039). In addition, trends towards higher transcriptional levels of cytokines in peripheral blood were observed more often in overweight women than in non-overweight women. In this study of 55 women with breast cancer, nine (16%) with metastases, diet became more pro-inflammatory with fewer antioxidants during the chemotherapy. Briefly, we have shown that chemotherapy is critical for high-risk overweight women due to their reduced intake of antioxidant nutrients, generating greater inflammatory and oxidative stress profiles, suggesting the adoption of healthier dietary practices by women with breast cancer throughout their chemotherapy.

## 1. Introduction

Inflammation and oxidative stress can influence cancer and both phenomena are related to one another [1,2,3,4,5,6,7,8,9]. Chronic inflammation exerts strong influence in the tumor microenvironment, impacting the processes of cancer progression and metastasis [10,11,12]. Cytokines such as Interleukin 6 (IL-6), Tumor Necrosis Factor alpha (TNF-α), Interleukin- 1β (IL-1β) and Interleukin 10 (IL-10), which are released peripherally and in the tumor microenvironment, can act locally in reciprocal signaling interactions [5,11,13,14,15,16].

IL-6 is a crucial proinflammatory cytokine produced at the inflammation site [11] and high IL-6 concentration in plasma and tissue may grant a worse prognosis to patients with breast cancer (BC) [5,11]. TNF-α is a cytokine capable of promoting tumor growth and migration, as high levels in the peripheral blood of cancer patients are associated with more advanced tumor stages and lymph node metastases [5]. IL-1 is produced mainly by mononuclear phagocytes and macrophages [14], while IL-1β consists of the main secreted biologically active form [15]. The presence of IL-1β in the tumor microenvironment is correlated with the greater occurrence of metastases, cell proliferation and angiogenesis [14]. IL-10 is considered a cytokine with an anti-inflammatory profile, since it inhibits gene expression and the production of inflammatory mediators by T cells and macrophages [16]. However, in some types of cancer, IL-10 can perform both anti-tumor and pro-tumor activities [16].

Reactive oxygen species (ROS) are produced by enzymatic and non-enzymatic systems and perform significant roles in cell physiology and pathophysiology. Although physiological concentrations are essential to ensure cell survival, overproduction of ROS can lead to a pro-oxidant environment; oxidative stress is considered an important factor in developing various diseases, e.g., neurodegenerative diseases, cardiovascular disorders and cancer [17]. Oxidative stress levels can change based on the tumor’s presence, progression and treatments, including surgery, radiation and chemotherapy. Due to the critical role of oxidative stress mechanisms in the treatment of BC and potentially of metastases, it has been proposed that oxidative stress may be particularly important in cancer prognosis and may be a risk factor. However, the epidemiological literature in this area is limited [18,19].

Antioxidant nutrients are molecules that can inhibit, chelate or eliminate ROS. The diet is the main external source of regulation of the plasma redox state, but the molecular mechanisms are still being studied. The total antioxidant capacity (TACd) has been shown to be a very interesting and useful way to estimate the total antioxidant content in the diet, as it takes into account all the bioactive compounds present in the diet and the synergistic effects between them in combating ROS [6]. Lipid peroxidation, caused by ROS, may be associated with survival after the diagnosis of BC [19]. According to Carioca et al. [20], adiposity and clinical staging of patients with BC are correlated with oxidative stress.

Food consumption can influence the inflammatory process, which can be quantified by the Dietary Inflammatory Index (DII^®^), based on literature associating dietary factors with the expression of six inflammatory markers: IL-1β, IL-4, IL-6, IL-10, TNF-α and C-reactive protein [21]. A typical pro-inflammatory diet has high amounts of saturated fat, refined carbohydrates, red meat, protein, cholesterol, and trans fat, while being low in fruits and vegetables [22,23]. An example of a diet with an anti-inflammatory profile is the opposite of this: foods that are nutrient-dense, colorful and full of flavor (e.g., rich in fruits, vegetables, fish and whole grains, with low consumption of butter and red meat, and moderate consumption of olive oil and alcohol [24]).

There is evidence that the high expression of inflammatory markers correlates with a greater risk of developing cancer and a worse prognosis for cancer patients [22,25,26,27]. Likewise, there seems to be an association between high levels of ROS and carcinogenesis, but the role of antioxidants in advanced cancer is still controversial, because during treatment, for the tumor to regress, therapies that induce ROS production are used [28,29,30]. Thus, mathematical models have been proposed to improve comprehension of the sophisticated functioning of cancer redox biology and to put forward new insights [31].

Chemotherapy (CT) is indicated for patients with more advanced, later-stage BC. CT may be administered before (neoadjuvant) or after (adjuvant) surgery to reduce or remove the tumor mass. Drugs such as taxanes (docetaxel and paclitaxel), vinca alkaloids (vinblastine and vincristine) and antimetabolites (anti-folates) stimulate the release of cytochrome c from mitochondria, inducing cell death, and intervening with the electron transport chain, culminating in the production of superoxide radicals [32]. Platinum complexes (e.g., cisplatin, carboplatin and oxaliplatin) and anthracyclines (e.g., doxorubicin, epirubicin and daunorubicin) generate extremely high levels of ROS [32].

So far, there are no specific recommendations for patients with BC during the CT process regarding antioxidant and inflammatory dietary parameters. Many of the studies focus only on the role of inflammation or oxidative stress for the risk of developing a tumor, without paying attention to the investigation of these factors working together in women with BC during CT, thus making our study unprecedented. Thus, our objective was to associate inflammatory and antioxidant markers (endogenous and exogenous) with nutritional status in overweight women with BC on CT, in addition to observing the occurrence of metastases three years after the end of treatment.

## 2. Materials and Methods

### 2.1. Study Design, Sample Size and Eligibility Criteria

This prospective study was conducted in 2014–15, at a Clinical Hospital in Uberlândia, MG, Brazil, after approval by the Ethics Committee on Human Research (protocol 721.977/14, addendum n°. 1.111.998/15 721.977/14). The entire study was developed according the standards of the Declaration of Helsinki and Resolution CNS 466/12.

The follow-up time for women with BC in CT varied based on the chemotherapy regimen; i.e., from about four to six months. The evaluations were carried out three times: T0, after the infusion of the first CT cycle; T1, after infusion of the intermediate cycle; and T2, after the infusion of the last CT cycle.

G*Power software, version 3.1 was used to calculate the sample size [33], taking into account the F ANOVA test of one-way repeated measures with an intermediate effect size of 0.25, alpha level of 0.05, 95% power and three measurements. With an adjustment of 20% for possible losses to follow up, a minimum of 52 women was required at the initial time (T0).

**Figure 1:**
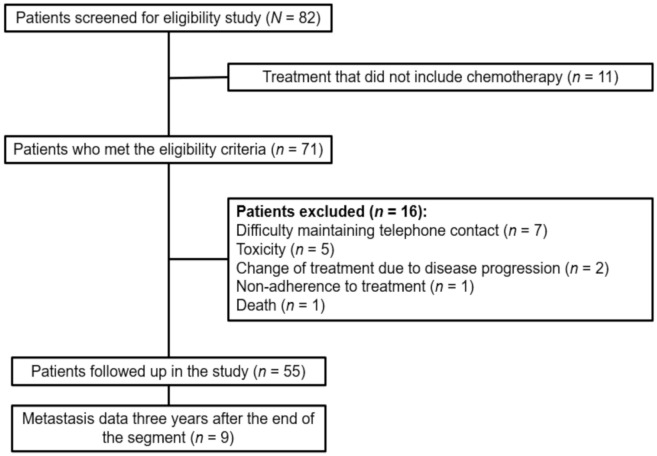
Diagram reporting the number of women with breast cancer screened, approached and recruited during a study at a university hospital in Uberlândia, Minas Gerais, Brazil, 2014–2015 (*n* = 55).

Inclusion criteria were defined as being a female ≥18 years, with BC and in the first cycle of first-line CT at T0. Figure 1 shows the number of women screened, approached and enrolled during the study.

### 2.2. Data and Biological Material Collections

The socioeconomic, therapeutic and clinical data of the study participants were obtained from interviews and medical records.

At time T2, 5 mL of peripheral blood was collected from each patient for analysis of inflammatory biomarkers and those related to the redox state. Such collection occurred after the infusion of chemotherapy in T2, with no other requirement, including fasting.

### 2.3. Extraction of Total RNA from Peripheral Blood, Reverse Transcription and qPCR

RNA was extracted from peripheral blood using Trizol LS reagent (Invitrogen, Life Technologies, Carlsbad, CA, USA) and following the manufacturer’s recommendations. The extraction product was quantified and evaluated by means of a spectrophotometric reading at 260 nm by NanoDrop (ThermoFisher Scientific). The extraction product was also subjected to agarose gel electrophoresis (1.5% agarose and 0.5 μg/mL ethidium bromide) made in TBE buffer (45 mM Tris-borate, pH 8.3 and 1 mM EDTA). After 1 h at 100 volts, electrophoretic profile was visualized in UV light and documented in ImageSystem - VDS (Amersham Biosciences) to evaluate the quality of RNA extraction.

For reverse transcription, 1 μg of total RNA, 10 U of RNase inhibitor (Invitrogen), 40 U of MMLV-RT (Amersham Biosciences), 1X of MMLV-RT buffer (Amersham Biosciences), 200 μM dNTPs (dGTP, dATP, dTTP and dCTP) and 126 pmoles of random primers were used. The solution was incubated in PTC-100 thermocycler (MJ Research) at 37 °C for 1 h and heated at 95 °C for 5 min. Control reactions (without RNA) were performed to verify possible exogenous contaminants. The cDNA was stored at −20 °C for further amplification.

The quality of the cDNA was assessed by amplification of a 536 bp fragment of the constitutive gene β-2-Microglobulin (*B2M*) flanked by the oligonucleotides: 5′-AGCAGAGAATGGAAAGTCAAA 3′and 5′ TGTTGATGTTGGAGAGAGAA 3′. For this reaction, 1X PCR buffer (20 mM Tris-HCl—pH 8.0, 0.1 mM EDTA, 1 mM DTT, 50% *v/v* glycerol) (Invitrogen), 200 μM dNTPs (Invitrogen), 5 pmoles primers (Invitrogen), 1 U Platinum^®^Taq DNA polymerase (Invitrogen), and 4 mM MgCl2 (Invitrogen) were used. Each reaction comprised the following steps: 95 °C for 4 min, 35 cycles of 94 °C for 40 s, 59 °C for 50 s, 72 °C for 50 s and 72 °C for 10 min in PTC-100 thermal cycler (MJ Research Inc.). The product of the reaction was analyzed by agarose gel electrophoresis.

In order to establish the transcriptional profile of biomarkers IL-1β, IL-6, IL-10 e TNFα, pairs of oligonucleotides were designed for each of the gene sequences and for the reference gene *B2M*. Oligonucleotides flanking fragments in sizes of 50 to 150 base pairs and that were considered viable for amplification standards according to Primer Express version 3.0 software (Applied Biosystems) were used. The specificity of the oligonucleotides was first evaluated in conventional PCR, to be used later in real-time PCR (qPCR).

The transcriptional quantifications of the target genes (IL-1β, IL-6, IL-10 e TNFα) in relation to the endogenous gene (*B2M*) were estimated using qPCR from the obtained cDNA. Each sample was analyzed in duplicate in a reaction containing 5.0 µL of Power SYBR Green PCR Master Mix (Applied Biosystems), and 0.5 µM of primers, according to the use of the manufacturer. Standard relative curves were created to validate the 2^−∆∆Cq^ comparative method.

### 2.4. Determination of Plasma Redox Status Biomarkers

For the reduced glutathione (GSH) (U/mg protein) quantification, 150 µL of plasma samples were added to 150 µL of metaphosphoric acid (ratio 1:1). The samples were centrifuged at 7000× *g* for 10 min at 4 °C and the supernatant was used for the measurements. A standard curve of GSH (0.001–0.1 mM) was created to quantify GSH in the samples using a linear regression. GSH reacts with ortho-phthalaldehyde (OPT 1 mg/mL methanol) diluted in sodium phosphate monobasic buffer (0.1 M) and EDTA (0.005 M). This mixture was incubated in the dark at room temperature for 15 min, and the fluorescence read at 350 nm (excitation) and 420 nm (emission) in a spectrophotometer [34].

The glutathione reductase (GR) (U/mg protein) assay to evaluate the enzymatic activity was determined from the consumption of nicotinamide (NADPH) using GSSG as a substrate. Plasma samples were mixed with 200 mmol L^−1^ sodium phosphate buffer pH 7.5 containing 6.3 mmol L^−1^ EDTA to inhibit metal-dependent enzymes, such as metalloproteases, 10 mmol L^−1^ oxidized glutathione and 3 mmol L^−1^ of NADPH. The decay of the NADPH concentration was quantified for 10 min at 340 nm in a spectrophotometer [34].

Glutathione peroxidase (GPx) (U/mg protein) activity was determined from the reduction of NADPH by GR. Plasma samples were incubated with GPx buffer (100 mM potassium phosphate, pH 7.7, containing 1 mM EDTA), 40 mM sodium azide, 100 mM GSH diluted in metaphosphoric acid 5%, GR diluted in GPx buffer, 2 mM of NADPH diluted in 5% sodium bicarbonate, and 0.5 mM tert-butyl. The decay of the NADPH concentration was evaluated for 10 min at 340 nm in a spectrophotometer [34].

Superoxide dismutase (SOD) (U/mg protein) activity is based on the auto-oxidation capacity of pyrogallol. The inhibition of pyrogallol auto-oxidation occurs in the presence of SOD, whose activity can be indirectly analyzed in a spectrophotometer at 420 nm, in a medium containing 50 mM Tris buffer with 1 mM EDTA pH 8.2, 80 U/mL of catalase, pyrogallol 0.38 mM and 15 µl of sample. A calibration curve was created using purified SOD as a standard. The 50% inhibition of pyrogallol auto-oxidation is designated as a unit of SOD [34].

The catalase (U/mg protein) activity was determined based on monitoring the decomposition of H_2_O_2_ at 240 nm, in a reaction medium containing 20 mM H_2_O_2_, 10 mM phosphate buffer (pH 7.0) and 10 µL of sample. The kinetic reading was executed at a wavelength of 240 nm for 10 min in a spectrophotometer [34].

### 2.5. Dietary Parameters

#### 2.5.1. 24-h Dietary Recall

The evaluation of the diet was carried out using data collected from the telephone administration of three 24-h dietary recalls (24HR) on non-consecutive days, one on the weekend, during each time of the study, totaling nine 24HR per women. Telephone calls were made in the interval between cycles (Δt = 21 days) in the second week after the administration to avoid the acute adverse effects of the treatment. This method identifies and quantifies all foods and beverages consumed the day before the interview.

The data, provided in home measurements on the 24HR, were converted into units of measurement (grams or milliliters) by the software Dietpro^®^ version 5.7. For the quantification of nutrients, the software Nutrition Data System for Research (NDSR) was used, adopting the international reference United States Department of Agriculture [35] and, for those foods not found in this table, the Brazilian Composition Table was used [36].

Due to the intrinsic variability of food consumption, the values for energy and nutrient consumption were deattenuated, i.e., adjusted for intra-individual variability, following the methodology of Nusser et al. [37] and using the PC-Side software (Department of Statistics, Iowa State University, Ames, Iowa, USA). Subsequently, in order to correct nutrient estimates, these were adjusted by the average energy of the sample, using the residual method [38].

Sixteen antioxidant nutrients were counted: vitamin A, beta-carotene, vitamin D, vitamin C, magnesium, iron, zinc, selenium, omega-6, omega-3, isoflavones, copper, glutamic acid, glycine, lutein + zeaxanthin and manganese.

#### 2.5.2. Calculation of TACd and DII Scores

TACd and DII^®^ were calculated from data obtained in the nine 24HR periods for each patient in CT. Regarding TACd, the table of antioxidant foods [39], developed to evaluate the total antioxidant content of complex diets, was used to obtain the FRAP (Ferric Reducing Antioxidant Power) values of foods. This contains more than 3100 foods, drinks, spices, herbs and supplements used worldwide.

The relationship between diet and oxidative stress has also been considered, with Total Dietary Antioxidant Capacity (TACd) being a method of measuring antioxidant intake. The calculation of TACd is basically the sum of the antioxidant power of all foods shown in the diet and the synergistic effects between them, since cooperation between several antioxidants affords greater protection against reactive species [40]. Therefore, the analysis becomes more robust when compared to the study of micronutrients known to be antioxidants separately [39].

However, some specific foods present in the diet of the Brazilian population are not included in the table of antioxidant foods developed by Carlsen et al. [39]. Thus, other literature was searched to determine the antioxidant power of the following items: carioca beans, coffee, guarana soda, toasted mate tea, açai, coconut, acerola, pequi and jaboticaba [41,42,43]. It is important to consider that the table presented by Carlsen et al. includes coffee; however, in our study, we chose to use the FRAP value of a national coffee, as it is a food with a high antioxidant power and very frequent in the consumption of our population.

DII scores were calculated according to the method of Shivappa et al. [21]. A higher DII score demonstrates a pro-inflammatory diet while a lower score demonstrates an anti-inflammatory diet [21]. In our study, DII was calculated with a total of 28 food parameters: vitamin B12, vitamin B6, beta-carotene, carbohydrate, cholesterol, energy, fat, fiber, iron, magnesium, monounsaturated fatty acids (MUFAs) and polyunsaturated fatty acids (PUFAs), niacin, omega 3, omega 6, protein, riboflavin, saturated fat, selenium, thiamine, vitamin C, vitamin D, vitamin E, zinc, isoflavone, trans fat, vitamin A, and folic acid.

#### 2.5.3. Assessment of Nutritional Status

Following the WHO protocol, anthropometric [44] current weight and height were measured to calculate the body mass index (BMI = weight [kg]/height [m]^2^), which was classified as recommended by WHO [45] for adults (age > 20 years and < 60 years) and by Lipschtz [46] for elderly women (≥ 60 years).

To investigate the association of overweight with inflammatory and antioxidant markers, women were divided into two groups: non-overweight (adult women with a BMI < 25 kg/m^2^ and elderly women with a BMI ≤ 27 kg/m^2^) and overweight (adult women with a BMI ≥ 25 kg/m^2^ and elderly women with a BMI > 27 kg/m^2^).

### 2.6. Search for Medical Records after the End of the Segment

The medical records of the patients were researched in the Archive and Research Sector of the Clinic Hospital and in the Archive Sector of the Cancer Hospital maximum period until the completion of the writing of this study (three years). The data sought were those referring to metastasis after the end of the last chemotherapy cycle (T2).

### 2.7. Statistical Analysis

Statistical analyzes were conducted using the SPSS Program version 21.0. Normality tests were performed for the variables, considering Shapiro-Wilk, making it possible to define whether parametric or non-parametric tests would be performed. The one-way ANOVA test with repeated measures was chosen to search for the antioxidant nutritional profile and DII^®^ of women in the three evaluation time points (T0, T1 and T2). The Friedman test also was performed.

In order to correlate redox status markers with transcriptional levels of cytokines, the Generalized Linear Models (GLzM) test was performed. The Mann Whitney test was used to verify the difference between the means of two subgroups, non-overweight and overweight women. The 95% confidence interval and *p*-value <0.05 were determined for all tests.

## 3. Results

### 3.1. Characterization of the Study Population

The study was conducted with 55 women, with a mean age ± standard deviation (SD) of 51.5 ± 10.1 years. Table 1 presents clinical, hormonal and therapeutic characteristics of the study participants. Regarding hormonal characteristics, 61.8% (*n* = 34) are postmenopausal women. As for the tumoral aspects, 25.4% (*n* = 14) were classified as clinical stage IIB; 58.2% (*n* = 32) were classified in histological grade G2. Regarding the molecular subtype, 58.1% were classified as luminal A/luminal B subtypes, phenotypes considered less aggressive and more responsive to treatment.

Table 2 shows the classification of the nutritional status by BMI of participants in three moments of the study (T0, T1 and T2).

### 3.2. Throughout the CT There Is a Decrease in the Intake of Antioxidants and an Increase in the DII^®^ Score

The consumption of antioxidants by the women in the study is presented in Table 3, in which a significant decrease in the intake of all antioxidant nutrients was seen at the end of treatment (T2) in comparison to the beginning (T0), except copper, in which the observed difference was between T2 and T1.

Regarding the consumption of the anti-inflammatory nutrients, the Friedman test showed that the median for consumption of lutein + zeaxanthin, manganese and daidzein differed between the three times (T0, T1 and T2). In addition, the median for consumption of total vitamin A, beta-carotene, vitamin C, isoflavones and genistein differed between T2 and the other times, but it did not differ between T0 and T1.

Furthermore, it was found that the median consumption of vitamin D, magnesium, iron, selenium, omega-6, omega-3, glutamic acid and glycine differed between T0 and the other times, but between T1 and T2 it did not differ. The multiple comparison test showed that the median for copper consumption differed only between T1 and T2.

Results of one-way repeated measures ANOVA showed that there is an effect of the time factor on the mean zinc consumption [F(1.71, 92.52) = 5.39; *p* = 0.009]. Sidak’s post-hoc showed that the mean consumption of this nutrient differed between T0 and the other times, but between T1 and T2 it did not differ.

Table 4 shows the results of the variation in the DII throughout CT. Differences between CT times revealed an increase in the consumption of a pro-inflammatory diet (*p* < 0.001).

### 3.3. Antioxidant Markers Are Associated with the Transcription of Inflammatory Markers

From the peripheral blood collected at the end of the CT (T2), the RNA was used to characterize the transcriptional levels of the cytokines IL-1β, IL-6, IL-10 and TNF-α, and the plasma was used to assess GSH levels and activity of GR, GPx, catalase (CAT) and SOD enzymes. Supplementary Tables S2 and S3 show the levels found through the median and the 25th and 75th percentiles.

The association of redox status markers with inflammatory markers was evaluated. The median of antioxidant markers was used as a criterion for dividing into two categories: above the median was considered higher antioxidant level and below the median was considered lower antioxidant level. It is relevant to notice that a reference value for such enzymes in plasma of CT patients (T2) is unknown. Table 5; Table 6 refer to the association between the plasma levels of redox status markers and the transcription of inflammatory cytokines.

Results with values of *p* < 0.05 were found for the association of GR with IL-1β (*p* < 0.001); GPx with IL-1β (*p* = 0.04), IL-10 (*p* = 0.001) and TNF-α (*p* = 0.001); and GSH with IL-10 (*p* = 0.045) and TNF-α (*p* = 0.047).

An association was found between lower GR levels and higher transcription of IL-1β (*p* < 0.001), IL-6 (*p* = 0.74), IL-10 (*p* = 0.09) and TNF-α (*p* = 0.08).

### 3.4. Overweight Women with BC Undergoing CT Have Lower Levels of Antioxidant Markers and Higher Levels of Inflammatory Markers When Compared to Non-Overweight

**Figure 2:**
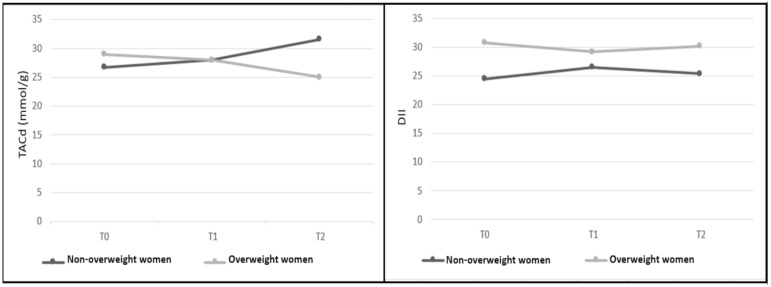
Association of overweight with the Total Dietary Antioxidant Capacity without coffee (TACd) and with the Dietary Inflammatory Index (DII) at T0, T1 and T2 (*n* = 55). TACd, Total Dietary Antioxidant Capacity without coffee; DII, Dietary Inflammatory Index; T0, Period after the first chemotherapy cycle; T1, Period after intermediate chemotherapy cycle; T2, Period after the last chemotherapy cycle. Dark gray shows means for non-overweight women and light gray shows means for overweight women. Mann Whitney test.

Figure 2 shows that the TACd is lower and the DII is higher in the group of overweight patients than in the group of non-overweight patients at the end of the follow-up.

**Figure 3:**
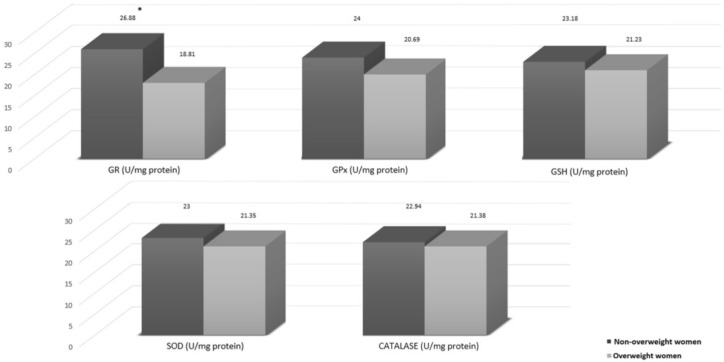
Association of overweight with plasma markers related redox status in T2 (*n* = 43). GR, Glutathione Reductase; GPx, Glutathione Peroxidase; GSH, Reduced Glutathione; SOD, Superoxide Dismutase; CAT, Catalase. * Value of *p* = 0.039. Dark gray shows means for non-overweight women and light gray shows means for overweight women. Mann Whitney test.

**Figure 4:**
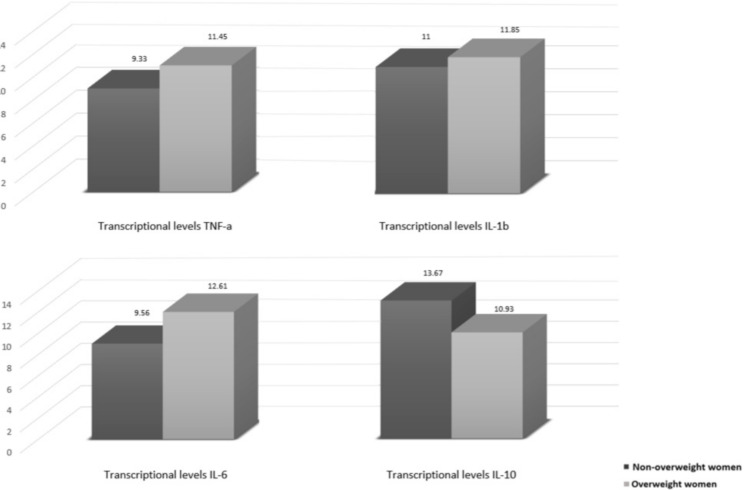
Association of overweight with inflammatory markers in T2. IL-1β, Interleukin-1β (*n* = 22); IL-6, Interleukin-6 (*n* = 22); IL-10, Interleukin-10 (*n* = 23); TNF-α, Tumor Necrosis Factor α (*n* = 20). Dark gray shows means for non-overweight women and light gray shows means for overweight women. Mann Whitney test.

Plasma markers related to the redox status in T2 are shown in Figure 3, and there is a notable difference between the non-overweight group (higher levels) and overweight group (lower levels), highlighting the plasma levels of GR that were significantly lower in overweight women than in non-overweight women (*p* = 0.039). In addition, trends towards higher transcriptional levels of cytokines in peripheral blood were noticed in overweight women compared to non-overweight women (Figure 4).

### 3.5. After the End of the Follow-Up, 16.4% of the Patients Developed Metastases in Three Years

Three years after the end of patient follow-up, the medical records of the studied women were searched in the archive and research sector of the university hospital of Uberlândia. In these searches, nine cases of post-treatment metastases were found, all distant. In order to characterize TACd and DII specifically for these cases (16.4% of patients), Supplementary Table S4 presents their respective values. Among these nine patients with metastases, five (55.5%) were from the overweight group and 4 (44.5%) were from the non-overweight group.

## 4. Discussion

The antioxidant and inflammatory dietary parameters changed throughout CT, i.e., a reduction in the intake of antioxidant nutrients and an increase in DII was found. In addition, excess weight was associated with a lower TACd in T0 and T2 and a lower GR level in T2; and the search for medical records three years after the end of T2 demonstrated the occurrence of 16.4% of metastases. In this sense, our study improves the information available at this critical moment for these overweight women, suggesting the importance of adopting healthier eating practices by women with BC throughout the CT. The most common BC subtype is ductal carcinoma [47]. In the present study, among the 55 participating women, 53 women had ductal carcinoma, i.e., 96.4%, reflecting the high occurrence of it in the general population.

Regarding the TACd, a study of more than 3000 women found an association between lower risk of BC in postmenopausal women with moderate and high TACd when compared to those with lower TACd. [29]. However, in our study, the TACd values did not differ between times (*p* = 0.60). Because of the high proportion of antioxidants in coffee concerning the total antioxidants ingested, and because of doubts regarding the amount of coffee polyphenols absorbed and with a real systemic role, we opted to remove the effect of coffee from the TACd value. This eliminates outlier values that could confound results, as demonstrated by other studies [48,49]. Thus, the Friedman test showed that the TACd without coffee differs between times (X2(2) = 7.89; *p* = 0.02), and the multiple comparison test showed that the TACd without coffee differs only between T0 and T2, corroborating the results already presented by our research group and by Rockenbach and collaborators [50], which reveal a negative impact of CT on diet quality [51,52] and on the intake of micro and macronutrients [51,52].

A diet with a lower DII score can improve levels of anti-inflammatory cytokines, and may reduce the risk of negative outcomes [21]. Shivappa et al. [25], in a prospective study with postmenopausal obese women, found an association between higher DII scores and increased risk of BC. Thus, the increase in DII scores over the CT course, as found in the present study, reflects the fragile condition of BC patients. Besides that, it may reflect the search for specific food groups [52] or types of food [53], often as a result of the alteration of food hedonism throughout CT [53].

Our data point to an association between lower GR levels and higher transcription of IL-1β (*p* <0.001), IL-6 (*p* = 0.74), IL-10 (*p* = 0.09) and TNF-α (*p* = 0.08). Nikulina et al. [54] state that the formation of ROS, nitric oxide (NO) and its derivatives can occur under IL-1β induction, i.e., this interleukin plays a significant role in the redox imbalance during chronic inflammation. Thus, our results are in agreement with these authors.

Through previous studies, it is known that GSH modulates NO synthesis in a pre- and post-transcriptional way, because low levels of this tripeptide lead to oxidative activation of the main transcription factor related to inflammation, called NFκB, and influences the enzymatic activity of induced nitric oxide synthase (iNOS), factors that are closely related to oxidative stress [54]. In our study, the association between lower GSH levels and higher transcription of IL-6 (*p* = 0.79), IL-10 (*p* = 0.045) and TNF-α (*p* = 0.047) was found. Weight gain and obesity are among the most relevant predictive factors for the risk of estrogen-dependent BC in postmenopausal women [55,56]. The high BMI favors insulin resistance and is significantly correlated with an increased risk of BC development and progression [55,57]. Regarding CT, studies indicate that weight gain is common among women in CT [58,59,60]. Changes in weight and diet may not be expected by women undergoing CT, causing concerns and anxieties that could be prevented by adequate guidance in the post-diagnosis phase [59].

Obesity can promote inflammation in the adipose tissue, leading to increased PGE2 production, which facilitates tumor promotion and progression [61]. Liu et al. [62] observed in peripheral blood of BC patients that the mRNA expression levels of IL-10 in non-overweight patients were 2.8 times higher than in overweight patients. Furthermore, when compared with obese, the IL-10 levels of the non-overweight group were 3.5 times higher (*p* < 0.05). In the present study, a higher transcriptional level of IL-10 was also found in the non-overweight group, and this result is likely to be linked to the anti-inflammatory profile that this cytokine can play [16].

There is evidence that a better quality diet seems to be related to lower levels of chronic inflammation and, consequently, with better disease-free survival [63,64,65]. This present study identified that women who presented post-treatment metastasis had a lower median TACd than the others. The alternative hypothesis would be that those women with post-treatment metastasis, in comparison to women without metastasis, would present higher TACd values, since a more antioxidant diet could negatively interfere with the effect of chemotherapy, considering that tumor cell death by this treatment is due to high ROS production [66,67]. However, the isolated analysis of TACd does not allow us to assess the treatment’s effectiveness, since it is not a known value according to which the supply of antioxidants would have a real effect on the ROS reduction and the non-apoptosis of tumor cells. It is important to note that proper nutrition can contribute to coping with diseases.

The controversial role played by antioxidants in cancer has persisted for decades. Although, prolonged exposure to ROS can inevitably induce long-term resistance to chemotherapy [68], currently the understanding of ROS’s subtle role in redox signaling and tumor proliferation, progression and metastasis, as well as molecular and cellular mechanisms, is still limited and needs to be further studied [69]. A comprehensive study of the changes that occur in the antioxidant pathways in cancer cells would bring considerable insights into understanding their unfolding. Thus, care should be taken when using dietary antioxidant supplementation, and enzymes from antioxidant pathways may be targets for chemoprevention; even the kinetics and time of exposure to ROS by CT are very different from those seen in primary prevention [70].

A better understanding of the tumor microenvironment is crucial for the development of better cancer treatments. ROS homeostasis’s dynamic maintenance is necessary for the induction of BC stem cell phenotype in response to hypoxia or cytotoxic chemotherapy [71].

The high control of the redox status by tumor cells is an important mechanism of resistance to chemotherapy and radiotherapy. Studies indicate that tumor cells with greater malignancy characteristics express a more significant amount of enzymes responsible for antioxidant defenses [72,73,74]. Resistance to multiple drugs is related, among other factors, to the expression of proteins involved in the greater ability to withdraw ROS from the cell interior [72]. Canli et al. [2] identified crosstalk between ROS molecules derived from inflammatory cells, oxidative damage to DNA and TNF-α-mediated signaling, promoting a pro-tumor and pro-metastatic microenvironment. Thus, as we could verify in our study, the ROS’s role in cancer is complex and comprehensive. The scarcity of study models in mice to monitor the tumor production of volatile ROS and to identify the cells most affected by ROS in vivo is a challenge for the expansion of research in this sense [75].

## 5. Conclusions

From the analyzes performed, a reduction in the intake of antioxidant nutrients and an increase in the DII throughout the CT was observed. In addition, overweight was associated with lower TACd in T2 and lower GR level in T2, as well as a trend towards higher transcriptional levels of cytokines in peripheral blood. The search for medical records three years after the end of T2 demonstrated the occurrence of 16.4% of metastases. We cannot affirm that the occurrence of post-treatment metastases is related to the parameters investigated in our study, even though they are closely related in the literature. However, we identified that the diet has become more pro-inflammatory and with less supply of antioxidants. In addition, we verify plasma levels of antioxidants and a tendency to higher inflammation associated with worse nutritional status (overweight) suggesting the importance of the adoption of healthier practices by women with BC throughout CT.

## Figures and Tables

**Table 1:** Clinical, hormonal and therapeutic characteristics of women with breast cancer (*n* = 55).

Characteristics	*n* (%)
**Age (years) mean (SD, min-max)**	51.5 (29–66 ± 10.1)
**Marital Status**	
Single	9 (16.4)
Married	27 (49)
Widow	7 (12.7)
Divorced/Separated	6 (10.9)
Other	6 (10.9)
**Years of Schooling**	
<8 years	18 (32.7)
8 to 11 years	24 (43.6)
>11 years	12 (21.8)
NR	1 (1.8)
**Menopause**	
No	21 (38.1)
Yes	34 (61.8)
**Tumor Subtype**	
Ductal carcinoma	53 (96.4)
Lobular carcinoma	2 (3.6)
**Clinical Stage**	
0	1 (1.8)
IA	10 (18.1)
IIA	12 (21.8)
IIB	14 (25.4)
IIIA	6 (10.9)
IIIB	8 (14.5)
IV	1 (1.8)
NR	3 (5.4)
**Histological Grade**	
G1	7 (12.7)
G2	32 (58.2)
G3	12 (21.8)
NR	4 (7.3)
**Molecular Subtypes**	
ER−, PR−, HER2− and CK5/6+ and/or EGFR+	11 (20)
ER−, PR− and HER2+	7 (12.7)
ER+ and/or PR+, HER2− and Ki-67 < 14%	14 (25.4)
ER+ and/or PR+, HER2− and Ki-67 ≥ 14%	18 (32.7)
ER+ and/or PR+, HER2+	5 (9.1)
**Previous Hormonal Therapy**	
No	48 (87.3)
Yes	7 (12.7)
**Surgery**	
Radical Mastectomy	7 (12.7)
Conservative Surgery	25 (45.4)
Others	23 (41.8)
**Chemotherapy Protocol**	
AC → Docetaxel (T)	33 (60)
AC → Paclitaxel (P)	8 (14.5)
FAC	9 (16.4)
CMF	5 (9.1)

SD, standard deviation; G1, well-differentiated tumor (low grade); G2, moderately differentiated tumor (intermediate grade); G3, poorly differentiated tumor (high degree); ER, estrogen receptor; PR, progesterone receptor; HER2, human epidermal growth factor type 2 receptor; −, negative; + positive; CK, cytokeratin; EGFR, human epidermal growth factor receptor; Ki 67, Ki 67 antigen; NR, not registered; AC, Adriamycin + cyclophosphamide; FAC, 5-fluoracil, Adriamycin and cyclophosphamide; CMF, cyclophosphamide, methotrexate and 5-fluorouracil.

**Table 2:** Nutritional status of women with breast cancer on chemotherapy at a university hospital in Uberlândia, MG, Brazil, 2014–2015 (*n* = 55).

Nutritional Status	Age Group	T0		T1		T2	
		*n*	%	*n*	%	*n*	%
Low weight	29–59	1	1.8	1	1.8	0	0.0
	60–66	2	3.6	2	3.6	2	3.6
Eutrophy	29–59	12	21.8	12	21.8	13	23.6
	60–66	9	16.4	9	16.4	10	18.2
Overweight	29–59	9	16.4	9	16.4	10	18.2
	60–66	7	12.7	7	12.7	6	10.9
Grade I obesity	29–59	6	10.9	7	12.7	6	10.9
Grade II obesity	29–59	6	10.9	5	9.1	5	9.1
Grade III obesity	29–59	3	5.5	3	5.5	3	5.5

T0, Period after the first chemotherapy cycle; T1, Period after intermediate chemotherapy cycle; T2, Period after the last chemotherapy cycle.

**Table 3:** Nutritional antioxidant profile of women with breast cancer on chemotherapy at a university hospital in Uberlândia, MG, Brazil, 2014–2015 (*n* = 55).

Antioxidant Nutrients	T0		T1		T2		*p*
	Mean ± SD	Median (p25–p75)	Mean ± SD	Median (p25–p75)	Mean ± SD	Median (p25–p75)	
Vitamin A (UI)	8521.6 ± 3932.9	7933.8 (5816.7–10280.3) ^a^	9791.7 ± 6248.8	7379.7 (5698.8–11217.7) ^a^	7517.5 ± 4550.8	6048.6 (4737.2–9799.5) ^b^	0.004 ᶷ
Beta-carotene (mcg)	4673.1 ± 1840.5	4552.7 (3340.0–5710.6) ^a^	4893.2 ± 3259.9	3852.0 (2903.4–5920.4) ^a^	3571.4 ± 2184.3	3050.8 (2285.8–4282.7) ^b^	<0.001 ᶷ
Vitamin D (mcg)	5.9 ± 4.0	4.8 (3.4–6.7) ^a^	4.1 ± 2.6	3.4 (2.0–5.0) ^b^	3.7 ± 2.4	3.1 (1.8–4.9) ^b^	<0.001 ᶷ
Vitamin C (mg)	130.3 ± 66.6	119.5 (83.8–159.2) ^a^	141.1 ± 66.8	123.5 (88.2–192.1) ^a^	80.4 ± 41.4	69.7 (51.2–101.2) ^b^	<0.001 ᶷ
Magnesium (mg)	216.4 ± 36.3	208.5 (191.6–227.6) ^a^	183.9 ± 35.9	175.2 (156.5–202.5) ^b^	179.0 ± 38.0	170.2 (156.6–195.2) ^b^	<0.001 ᶷ
Iron (mg)	10.3 ± 1.2	10.3 (9.3–11.0) ^a^	8.9 ± 1.5	8.9 (8.1–9.7) ^b^	9.0 ± 1.5	9.0 (7.8–9.9) ^b^	<0.001 ᶷ
Zinc (mg)	8.2 ± 1.2 ^a^	8.0 (7.3–8.7)	7.7 ± 1.2 ^b^	7.7 (6.5–8.5)	7.4 ± 1.5 ^b^	7.3 (6.5–8.4)	0.009 ᶿ
Selenium (mcg)	108.9 ± 55.6	96.1 (86.8–112.6) ^a^	93.2 ± 23.5	89.1 (77.1–103.8) ^b^	85.4 ± 12.2	85.7 (76.4–93.2) ^b^	<0.001 ᶷ
Omega-6 (g)	10.1 ± 1.4	10.2 (9.1–11.1) ^a^	9.2 ± 1.5	8.9 (8.0–10.2) ^b^	8.8 ± 1.1	8.6 (8.1–9.8) ^b^	<0.001 ᶷ
Omega-3 (g)	1.8 ± 0.3	1.7 (1.5–1.9) ^a^	1.3 ± 0.3	1.3 (1.2–1.4) ^b^	1.4 ± 0.3	1.4 (1.2–1.5) ^b^	<0.001 ᶷ
Isoflavones (mg)	0.9 ± 0.7	0.7 (0.5–1.1) ^a^	1.8 ± 5.2	0.7 (0.3–1.1) ^a^	0.2 ± 0.1	0.2 (0.1–0.3) ^b^	<0.001 ᶷ
Copper (mg)	1.2 ± 0.6	1.0 (0.9–1.3) ^a,b^	1.2 ± 0.5	1.1 (0.9–1.4) ^a^	1.2 ± 1.2	0.8 (0.7–1.3) ^b^	0.02 ᶷ
Glutamic Acid (g)	11.0 ± 0.7	11.0 (10.7–11.3) ^a^	9.9 ± 1.3	10.0 (9.0–10.5) ^b^	10.2 ± 1.1	10.3 (9.4–10.8) ^b^	<0.001 ᶷ
Glycine (g)	2.8 ± 0.1	2.9 (2.7–2.9) ^a^	2.5 ± 0.4	2.5 (2.2–2.8) ^b^	2.5 ± 0.5	2.4 (2.1–2.8) ^b^	<0.001 ᶷ
Lutein + Zeaxanthin (mcg)	5300.6 ± 3376.6	4519.8 (3325.3–5609.5) ^a^	2078.1 ± 1746.5	1769.1 (954.7–2222.1) ^b^	3835.1 ± 2854.2	3170.8 (1850.5–4776.2) ^c^	<0.001 ᶷ
Manganese (mg)	8.0 ± 4.6	6.9 (4.9–9.1) ^a^	17.1 ± 19.1	12.6 (6.6–19.3) ^b^	8.9 ± 19.1	4.2 (2.3–7.0) ^c^	<0.001 ᶷ
Daidzein (mg)	0.4 ± 0.6	0.2 (0.1–0.4) ^a^	0.4 ± 0.9	0.1 (0.1–0.3) ^b^	0.07 ± 0.03	0.07 (0.05–0.09) ^c^	<0.001 ᶷ
Genistein (mg)	0.3 ± 0.3	0.2 (0.1–0.4) ^a^	2.4 ± 11.8	0.1 (0.1–0.5) ^a^	0.04 ± 0.02	0.04 (0.03–0.05) ^b^	<0.001 ᶷ
TACd without coffee (escore)	7.4 ± 9.9	4.1 (2.6–7.6) ^a^	7.7 ± 10.9	4.1 (2.7–7.7) ^a.b^	4.4 ± 4.9	2.8 (1.8–5.2) ^b^	0.019 ᶷ

SD, standard deviation; T0, Period after the first chemotherapy cycle; T1, Period after intermediate chemotherapy cycle; T2, Period after the last chemotherapy cycle.; TACd, Total Dietary Antioxidant Capacity; ᶿ One-way ANOVA with repeated measurements + Sidak test; ᶷ Friedman + Multiple Comparison Test; Horizontal means/medians followed by different letters differed statistically according to the post-hoc test at the 5% probability level.

**Table 4:** Dietary Inflammatory Index (DII) by time of chemotherapy of women with breast cancer at a university hospital in Uberlândia, MG, Brazil, 2014–2015 (*n* = 55).

DII	T0		T1		T2		*p*
	Mean ± SD	Median (p25–p75)	Mean ± SD	Median (p25–p75)	Mean ± SD	Median (p25–p75)	
Score DII	0.04 ± 1.16	0.14 (−0.77–0.74) ^a^	0.79 ± 1.08	0.86 (−0.06–1.64) ^b^	1.78 ± 1.02	1.89 (1.17–2.42) ^c^	<0.001 ᶷ

DII, Dietary Inflammatory Index; SD, standard deviation; T0, Period after the first chemotherapy cycle; T1, Period after intermediate chemotherapy cycle; T2, Period after the last chemotherapy cycle; ᶷ Friedman + Multiple Comparison Test; Horizontal means/medians followed by different letters differed statistically according to the post-hoc test at the 5% probability level.

**Table 5:** Association between levels of redox status markers and transcriptional levels of inflammatory markers in women with breast cancer at the end of chemotherapy (T2).

Dependent Variables	Higher vs. Lower Antioxidant Level ^§^														
	GR					GPx					GSH				
	Lower Antioxidant Level Mean ± SD	Higher Antioxidant Level Mean ± SD	β	*p*	IC (95%)	Lower Antioxidant Level Mean ± SD	Higher Antioxidant Level Mean ± SD	β	*p*	IC (95%)	Lower Antioxidant Level Mean ± SD	Higher Antioxidant Level Mean ± SD	β	*p*	IC (95%)
**IL-1β**	19.27 ± 4.50	−9.57 ± 5.08	28.84	<0.001	13.14–44.54	−2.34 ± 4.38	12.04 ± 4.31	−14.37	**0.037**	−27.89–−0.87	−1.76 ± 5.99	11.47 ± 4.38	−13.23	0.144	−31.00–4.54
**IL-6**	17.48 ± 14.53	10.29 ± 13.14	7.19	0.744	−35.96–50.34	13.97 ± 13.21	13.81 ± 14.78	0.15	0.995	−43.84–44.14	17.26 ± 16.35	10.52 ± 13.78	6.74	0.789	−42.54–56.02
**IL-10**	275.90 ± 146.35	−107.86 ± 130.76	383.77	0.085	−52.44–819.99	−302.33 ± 133.62	470.37 ± 146.06	−772.70	**0.001**	−12014.80–−330.60	338.12 ± 163.48	−170.07 ± 138.93	508.20	**0.045**	10.21–1006.19
**TNF-α**	32.59 ± 15.72	−6.96 ± 13.48	39.56	0.082	−5.04–84.15	−24.46 ± 14.30	50.08 ± 15.20	−74.54	**0.001**	−119.73–−29.36	38.26 ± 17.15	−12.64 ± 14.32	50.91	**0.047**	0.61–101.21

IL-1β, Interleukin-1β (*n* = 22); IL-6, Interleukin-6 (*n* = 22); IL-10, Interleukin-10 (*n* = 23); TNF-α, Tumor Necrosis Factor α (*n* = 20); GR, Glutathione Reductase; GPx, Glutathione Peroxidase; GSH, Reduced Glutathione; SOD, Superoxide Dismutase; CAT, Catalase; SD, Standard Deviation; β, regression constant that represents the intercept of the line with the Y axis; IC, Confidence Interval. Generalized Linear Models (GLzM). Data adjusted for age, income and chemotherapy protocol. Values of *p* < 0.05 are shown in bold. ^§^ Higher antioxidant level was used as a reference category.

**Table 6:** Association between levels of redox status markers and transcriptional levels of inflammatory markers in women with breast cancer at the end of chemotherapy (T2).

Dependent Variables	Higher vs. Lower Antioxidant Level ^§^									
	SOD					CAT				
	Lower Antioxidant Level Mean ± SD	Higher Antioxidant Level Mean ± SD	β	*p*	IC (95%)	Lower Antioxidant Level Mean ± SD	Higher Antioxidant Level Mean ± SD	β	*p*	IC (95%)
IL-1β	5.81 ± 4.22	3.89 ± 3.31	1.920	0.724	−8.740–12.579	0.93 ± 3.33	8.77 ± 5.54	−7.835	0.293	−22.435–6.766
IL-6	21.02 ± 14.00	6.76 ± 10.47	14.255	0.432	−21.279–49.788	7.01 ± 11.05	20.77 ± 16.93	−13.755	0.552	−59.082–31.572
IL-10	−66.71 ± 141.21	234.75 ± 102.66	−301.469	0.099	−659.986–57.049	107.31 ± 111.76	60.72 ± 167.61	46.589	0.841	−407.473–500.652
TNF-α	0.32 ± 15.83	25.30 ± 10.67	−24.986	0.205	−63.645–13.672	12.85 ± 11.52	12.78 ± 17.80	0.071	0.998	−46.290–46.431

IL-1β, Interleukin-1β (*n* = 22); IL-6, Interleukin-6 (*n* = 22); IL-10, Interleukin-10 (*n* = 23); TNF-α, Tumor Necrosis Factor α (*n* = 20); GR, Glutathione Reductase; GPx, Glutathione Peroxidase; GSH, Reduced Glutathione; SOD, Superoxide Dismutase; CAT, Catalase; SD, Standard Deviation; β, regression constant that represents the intercept of the line with the Y axis; IC, Confidence Interval. Generalized Linear Models (GLzM). Data adjusted for age, income and chemotherapy protocol. ^§^ Higher antioxidant level was used as a reference category.

## Data Availability

Link repository: https://repositorio.ufu.br/handle/123456789/27102.

## Supplementary Materials

The following are available online at https://www.mdpi.com/2072-6643/12/11/3303/s1, Table S1: Sequence of primers used in qPCR; Table S2: Transcriptional levels of inflammatory markers analyzed in breast cancer patients on chemotherapy at a university hospital in Uberlândia, MG, Brazil, 2014–2015; Table S3: Plasma antioxidant markers in breast cancer patients undergoing chemotherapy at a university hospital in Uberlândia, MG, Brazil, 2014–2015; Table S4: Characterization of women who presented post-treatment metastasis—data obtained in 2019
