# Supplementary material for: Overweight Women with Breast Cancer on Chemotherapy Have More Unfavorable Inflammatory and Oxidative Stress Profiles
Source: Nutrients. 2020 Oct 28;12(11):3303. doi: 10.3390/nu12113303 (PMC7692181; doi:10.3390/nu12113303)
Supplement: Supplementary file 1 [file nutrients-12-03303-s001.pdf]

Table S1. Sequence of primers used in qPCR.

Primers	Sequence
B2M	*B2M* F: AGCAGAGAATGGAAAGTCAAA<br>*B2M* R: TGTTGATGTTGGATAAGAGAA
IL-1β	*IL-1*β F: ACAGGATATGGAGCAACAAGTGG<br>*IL1-β* R: GGGCTTATCATCTTTCAACACGC
IL-6	*IL-6* F: GATTCCAAAGATGTAGCCGCC<br>*IL-6* R: ATTTTCACCAGGCAAGTCTCCTC
IL-10	*IL-10* F: ACCCACTTCCCAGGCAACC<br>*IL-10* R: TGGCAACCCAGGTAACCCTT
TNFα	*TNFα* F: CAGGCGGTGCTTGTTCCT<br>*TNFα* R: GGGGTTCGAGAAGATGATCTGAC

F – primer forward; R – primer reverse.

Table S2. Transcriptional levels of inflammatory markers analyzed in breast cancer patients undergoing chemotherapy at a university hospital in Uberlândia, Minas Gerais, Brazil, 2014-2015.

Inflammatory Markers	N	Median (p25-p75)
IL-1β	22	1.11 (0.65-2.77)
IL-6	23	0.28 (0.14-0.72)
IL-10	23	1.06 (0.43-3.58)
TNFα	20	1.24 (0.48-3.98)

IL-1β, Interleukin-1β (n = 22); IL-6, Interleukin-6 (n = 22); IL-10, Interleukin-10 (n = 23); TNF-α, Tumor Necrosis Factor α (n = 20). Non-normal distribution.

Table S3. Plasma antioxidant markers in breast cancer patients undergoing chemotherapy at a university hospital in Uberlândia, Minas Gerais, Brazil, 2014-2015 (Median/mean and Percentiles/SD), n = 43.

Antioxidant Markers	Median (p25-p75)<br>Mean ± SD
GR	0.65 ± 0.49
GPx	6.51 ± 1.66
GSH	3.82 ± 0.81
CAT	0.13 ± 0.63
SOD[a]	0.10 (0.09-0.12)

GR, Glutathione Reductase; GPx, Glutathione Peroxidase; GSH, Reduced Glutathione; SOD, Superoxide Dismutase; CAT, Catalase; SD, Standard Deviation. [a] Non-normal distribution (the others have a normal distribution).

Table S4. Characterization of women who presented post-treatment metastasis - data obtained in 2019 (N = 9 for metastatic cases and N = 46 for non-metastatic cases).

Patient	TACd	DII
**ID1**	8.753	2.59
**ID2**	5.831	3.21
**ID3**	8.655	2.17
**ID4**	4.588	-0.41
**ID5**	10.196	1.13
**ID6**	2.784	1.87
**ID7**	4.435	2.19
**ID8**	2.047	1.89
**ID9**	11.477	-0.84

**Metastasis cases Median (p25-p75)**	5.83 (3.61 – 9.47)	1.90 (0.36 – 2.39)
**Non-metastasis cases Median (p25-p75)**	13.83 (7.93 – 21.22)	1.89 (1.21 – 2.45)

TACd, total antioxidant capacity of the diet; ID, identification; DII, dietary inflammatory index; metastasis cases, occurrence of metastases after T2 (n = 9); non-metastasis cases, no metastasis after T2 (n = 46).
